# Identification of co-regulated genes associated with doxorubicin resistance in the MCF-7/ADR cancer cell line

**DOI:** 10.3389/fonc.2023.1135836

**Published:** 2023-06-15

**Authors:** Ali Miri, Javad Gharechahi, Iman Samiei Mosleh, Kazem Sharifi, Vahid Jajarmi

**Affiliations:** ^1^ Department of Medical Biotechnology, School of Advanced Technologies in Medicine, Shahid Beheshti University of Medical Sciences, Tehran, Iran; ^2^ Human Genetic Research Center, Baqiyatallah University of Medical Sciences, Tehran, Iran; ^3^ Department of Bioinformatics, Institute of Biochemistry and Biophysics, University of Tehran, Tehran, Iran; ^4^ Anesthesiology Research Center, Shahid Beheshti University of Medical Sciences, Tehran, Iran

**Keywords:** breast cancer, chemoresistance, differentially expressed genes, gene co-expression network, doxorubicin (DOX)

## Abstract

**Introduction:**

The molecular mechanism of chemotherapy resistance in breast cancer is not well understood. The identification of genes associated with chemoresistance is critical for a better understanding of the molecular processes driving resistance.

**Methods:**

This study used a co-expression network analysis of Adriamycin (or doxorubicin)-resistant MCF-7 (MCF-7/ADR) and its parent MCF-7 cell lines to explore the mechanisms of drug resistance in breast cancer. Genes associated with doxorubicin resistance were extracted from two microarray datasets (GSE24460 and GSE76540) obtained from the Gene Expression Omnibus (GEO) database using the GEO2R web tool. The candidate differentially expressed genes (DEGs) with the highest degree and/or betweenness in the co-expression network were selected for further analysis. The expression of major DEGs was validated experimentally using qRT–PCR.

**Results:**

We identified twelve DEGs in MCF-7/ADR compared with its parent MCF-7 cell line, including 10 upregulated and 2 downregulated DEGs. Functional enrichment suggests a key role for RNA binding by IGF2BPs and epithelial-to-mesenchymal transition pathways in drug resistance in breast cancer.

**Discussion:**

Our findings suggested that *MMP1*, *VIM*, *CNN3*, *LDHB*, *NEFH*, *PLS3*, *AKAP12*, *TCEAL2*, and *ABCB1* genes play an important role in doxorubicin resistance and could be targeted for developing novel therapies by chemical synthesis approaches.

## Introduction

Breast cancer is the most common malignancy among women worldwide and is the second cause of cancer-related mortalities after lung cancer (1). In the United States alone, 268,600 new cases and 41,760 fatalities were reported for breast cancer in 2019 (2), accounting for greater than 30% of all new cancers and 15% of all cancer-related deaths. Breast cancer is distinguished by molecular, histological, and clinical characteristics, necessitating distinct clinical management strategies (3). Based on immunohistochemistry analysis, breast cancer can be classified into three distinct molecular subtypes, including estrogen or progesterone receptor positive (ER+/PR+), human epidermal growth factor receptor positive (HER2+), and triple-negative (TNBC) (4, 5). ER+/PR+ breast tumors have distinct gene expression signatures characteristic of ductal luminal cells of the breast and are accordingly subclassified into luminal A and luminal B subgroups with very different prognoses (6, 7). The luminal A subtype, for example, is characterized by a high expression of proliferative and cell cycle-related genes and a low proliferative rate (8). A high expression of Ki-67 and proliferating cell nuclear antigen and a high mutation rate of p53 are characteristics of luminal B subtypes (9, 10). TNBC or basal-like tumors are heterogeneous in gene expression profiles and can be categorized into multiple different subgroups (11).

Endocrine hormone therapies, including ovarian function suppression, selective estrogen receptor modulators, selective estrogen receptor down regulators, and aromatase inhibitors, are commonly used as primary systemic therapies in patients with ER+/PR+ breast cancer, complementing surgery (12, 13). HER2+ breast cancer, which accounts for ~20% of all breast cancer cases, benefits from therapies targeting the epidermal growth factor 2 (ERBB2 or HER2/neu) gene, such as anti-ERBB2 antibodies and tyrosine kinase inhibitors (13). However, TNBC, which represents about 15% of all breast cancer cases and is more common in premenopausal young women under 40, lacks effective targeted therapies and is unresponsive to current endocrine therapies (14). Chemotherapy, including doxorubicin/Adriamycin, paclitaxel, docetaxel, cyclophosphamide, and carboplatin, alone or in combination, is commonly used for neoadjuvant or adjuvant treatment of breast cancer, to downstage tumors or as a standard-of-care regimen for aggressive and early-stage disease (13, 15–20). Doxorubicin, an anthracycline chemotherapeutic agent, is still a first-line therapy for early-stage breast, ovarian, lymphoma, and leukemia cancers (21–24). However, the development of chemoresistance continues to be a significant clinical obstacle in treating breast cancer (15).

Chemoresistance refers to the ability of cancer cells to survive and proliferate despite exposure to high doses of chemotherapeutic agents, resulting in a lack of response or failure of the treatment. The most common routes for chemoresistance include over-expression of membrane efflux pumps, such as ATP-binding cassette (ABC) transporters, drug sequestration in lysosomes, alterations in drug metabolism, mutations or downregulation of drug targets, upregulation of cell cycle regulators and apoptosis inhibitors, activation of survival pathways, changes to cellular metabolism, mitochondrial alteration, and changes to the tumor microenvironment (25–33). Understanding the molecular mechanisms underlying doxorubicin resistance in breast cancer is crucial for developing effective strategies to overcome this resistance and improve patient outcomes.

Despite extensive research on doxorubicin resistance in breast cancer, there are still gaps in our knowledge regarding the specific genes and pathways involved in this process. In this study, we aimed to identify co-regulated genes associated with doxorubicin resistance in the MCF-7/ADR breast cancer cell line using gene co-expression network (GCN) analysis of publicly available microarray gene expression datasets (34). GCN analysis has been extensively used for the identification of genes and molecular pathways dysregulated in various cancers (35, 36), particularly those genes with uncertain significance in biological processes (37).

## Materials and methods

### Breast adenocarcinoma datasets

We searched the GEO database for breast cancer mRNA expression data related to doxorubicin or Adriamycin resistance, specifically focusing on datasets that included profiling of both MCF-7/ADR and normal parent MCF-7 cell lines for downstream analysis. Four human mRNA datasets (GSE5920, GSE87864, GSE24460, and GSE76540) were identified based on these criteria. After an initial analysis, we noticed that the GSE87864 and GSE5920 datasets were unsuitable for network analysis due to high heterogeneity among replicates and inconsistent patterns of results. The remaining two datasets comprised at least two cell lines (MCF-7/ADR and parent cell line MCF-7) and two experimental conditions (doxorubicin and no doxorubicin), and both were generated by the same Affymetrix platform (Affymetrix Human Genome U133 Plus 2.0 Array). **Table 1** shows detailed information about the analyzed datasets.

**Table 1:** The detailed characteristics of the datasets included in this study.

Country	Cancer Type	Samples		Platform	Dataset	DEGs
		MCF-7/ADR	MCF-7			
USA	Breast cancer	2	2	Affymetrix HG-U133A_2	GSE24460	1108
China	Breast cancer	3	3	Affymetrix HG-U133_Plus_2	GSE76540	3207

### Identification of dysregulated genes

**Figure 1:**
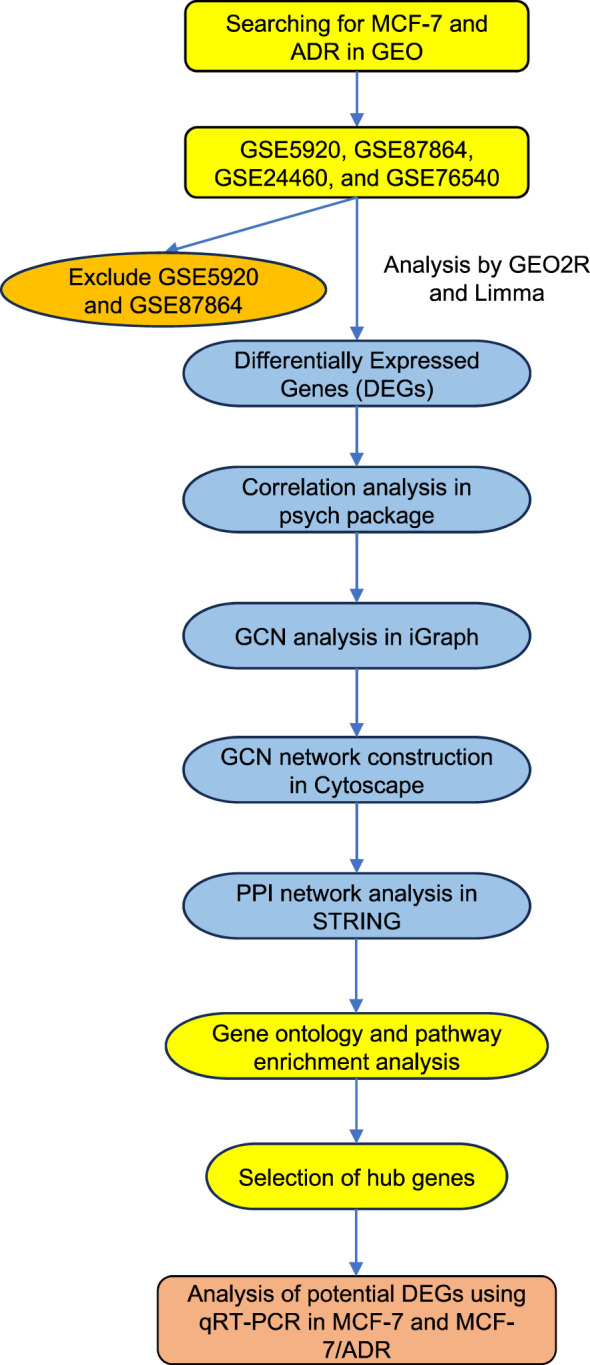
The diagram shows the overall analysis performed in this study. The mRNA expression datasets were retrieved from the GEO database by searching for the MCF-7 and ADR keywords. The candidate datasets were screened for the number of analyzed samples and the reproducibility of the expression data. Datasets with low sample numbers and inconsistent results were excluded from further analysis. DEGs, differentially expressed genes; PPI, Protein-Protein Interactions; GEO, Gene Expression Omnibus, GCN, Gene co-expression network.

Primary analyses were performed using the online GEO2R suite (http://www.ncbi.nlm.nih.gov/geo/geo2r). The GEO2R enables the comparison of samples in a GEO dataset and the identification of differentially expressed genes (DEGs) under a particular experimental condition. We ignored the probe sets without a gene symbol. The GEO2R build-in R package Limma was used to identify DEGs (38). The raw expression data were corrected for background noise and normalized using the Robust Multi-array Average (RMA) algorithm, which takes into account data quantiles to correct for array biases (39). DEGs were identified by comparing normalized expression data from MCF-7 and MCF-7/ADR cell lines and looking for a minimum |log2 fold change (FC)| > 1 and a Bonferroni corrected P-value < 0.05. Correlations (the Pearson method) between gene expression data were calculated using the psych package in R (40). Only correlations with |r| = 0.7 and P-value < 0.05 were considered for network construction. The resulting correlation matrix was used for network construction using the R package iGraph under the default settings (38). Gene clusters were generated using the clusterMaker plugin in Cytoscape based on the AutoSOME algorithm (41). Genes with the greatest betweenness and/or degree were chosen for further experimental validation, as previously described (42). **Figure 1** shows the details of the bioinformatic workflow employed to identify DEGs and the final hub genes.

### Functional annotation analysis

To assign specific functional roles to DEGs and to visualize them in the context of molecular pathways, we used a set of functional annotation and pathway inference tools. The Kyoto Encyclopedia of Genes and Genomes (KEGG) database was used for the functional annotation of DEGs. Gene ontology (GO) annotation allowed us to categorize DEGs into cellular components, molecular functions, and biological processes’ functional ontologies. Protein-protein interactions were inferred by the Search Tool for the Retrieval of Interacting Genes/Proteins (STRING, www.string-db.org/cgi/input.pl) considering all interaction sources. Interactions with a combined score > 0.4 were used for network visualization, and those with a score > 0.7 were kept for further analysis.

### Cell line characterization and authentication

The MCF-7 and MCF-7/ADR cell lines were obtained from the Iranian Biological Resources Center. The authenticity of cell lines was validated using Short Tandem Repeat (STR) DNA profiling. STR profiling was conducted using the AmpFlSTR Identifiler PCR Amplification Kit (Applied Biosystems, Foster City, CA, USA) according to the manufacturer’s instructions. STR profiles were evaluated using the GeneMapper ID software. The MCF-7/ADR cell line was further characterized based on cell morphologies and IC_50_ drug dosage.

### Cell culture

MCF-7 cells were cultured in Dulbecco’s modified Eagle’s medium (DMEM; Gibco source; DNA Biotech, Iran) supplemented with 100 units/mL penicillin-streptomycin and 10% FBS (BioIdea, Tehran, Iran) at 37 °C and 5% CO_2_. MCF-7/ADR cells were cultured in RPMI 1640 medium (Gibco source) containing 10% FBS and 13 μg/mL doxorubicin. The drug was omitted from the culture medium 48 h before the experiment.

### MTT assay to determine the lethal concentration (IC_50_) of doxorubicin

Cells were seeded at a density of 2×10^4^ cells/well in a 96-well culture plate and incubated at 37°C in a humidified environment containing 5% CO_2_ for 16 hours. Subsequently, doxorubicin was added at concentrations of 0, 5, 10, and 15 μg/mL after cells fully adhered to the plate. The experiment was conducted in triplicate, with each drug concentration assayed in at least three independent wells. The MTT assay was performed by adding 20 μL of 3-(4,5-dimethylthiazol-2-yl)-2,5-diphenyltetrazolium bromide reagent (5 mg/mL) to each well and the cells were incubated for an additional 4 h. After incubation, 100 μL of dimethyl sulfoxide (DMSO) was added to each well and the plate was shaken for 15 min to ensure the complete dissolution of cells. The optical density (OD) was measured at 492 nm using a microplate reader. Cell growth inhibition was calculated using the formula: inhibitory rate (percentage) = (1 - mean OD value in the experimental group/mean OD value in the control group) × 100. The IC_50_ value was estimated from the OD values and used to determine the doxorubicin cytostatic activity on MCF-7 and MCF-7/ADR cells.

### Total RNA extraction and quantitative real-time PCR analysis

Total RNA was isolated from MCF-7 and MCF-7/ADR cell lines using the Roche High Pure RNA Isolation Kit (Roche GmbH, Mannheim, Germany) according to the manufacturer’s instructions. The concentration and integrity of the extracted mRNAs were evaluated by a nanodrop spectrophotometer (Maestrogen, Taiwan) and agarose gel electrophoresis.

A total of 1 μg RNA was reverse transcribed in a 20 μL reaction mixture using the ExcelRT™ One-Step RT-qPCR Kit (SMOBIO, Taiwan) according to the manufacturer’s protocol. qRT–PCR reactions were carried out under the following cycling conditions: a denaturation step at 95°C for 10 min, followed by 40 cycles of 95°C for 15 s and 60°C for 1 min in an ABI Step One Plus™ Real-Time PCR System (Applied Biosystems). RT–PCR reactions were performed in triplicate. The cycle threshold (Ct) values for the target gene and internal control gene (GAPDH) were extracted and used to estimate gene expressions following the 2^-ΔΔCt^ method (43).

Primers used for qRT–PCR were designed using the Oligo7 software and searched against the human RefSeq database to verify their amplification specificities. **Table 2** shows the sequence of primers used for qRT–PCR.

**Table 2:** The sequence of primers used for quantitative real-time PCR (qRT-PCR) analysis of target genes.

Gene symbol	Primer sequence (5’ → 3’)
**ABCB1**	F: AACACCCGACTTACAGATGATG
	R: CTTCCAACCACGTGTAAATCCT
**AKAP12**	F: AAGTCATTGTCACAGAGGTTGGA
	R: CTCAGTGGGTTGTGTTAGCTCT
**CNN3**	F: CATCATCCTCTGCGAACTTATAAACA
	R: TTGCTTCGAATATGTCATGTGGC
**ESR1**	F: TGATGAAAGGTGGGATACGAAAAG
	R: GGTTGGCAGCTCTCATGTCT
**FXYD3**	F: TCCTTTCTACTATGACTGGCACA
	R: AGCTCCTCCACTCACTCATG
**VIM**	F: CCACGAAGAGGAAATCCAGGAG
	R: TACCATTCTTCTGCCTCCTGC
**LDHB**	F: GCGACTCAAGTGTGGCTGT
	R: GACTTCATAGGCACTTTCAACCAC
**MMP1**	F: GGACCAACAATTTCAGAGAGTACAA
	R: CCGATATCAGTAGAATGGGAGAGT
**NEFH**	F: GAGTGGTTCCGAGTGAGGC
	R: GCTCTGTGGTCCTGGCC
**PLS3**	F: TGGCAGCTGATGAGAAGATATACC
	R: TCCAGCTTCACTCAACGTTCT
**TCEAL2**	F: AGTCAGAGATGCAGGGAGGA
	R: TGCAGCCCTTGTTTCACTTTCT
**CTGF**	F: GTGTGCACCGCCAAAGATG
	R: GCTGGGCAGACGAACGT
**GAPDH**	F: GTATCGTGGAAGGACTCATGACC
	R: CAGTAGAGGCAGGGATGATGTTC

### Statistical analysis

All statistical analyses were performed using GraphPad Prism (Version 9, San Diego, CA). Statistically significant differences between the treatment groups were identified using the student t-test. Statistical data are presented as mean ± sd. A P-value less than 0.05 was considered statistically significant.

## Results

### Comparing gene expression profiles between MCF-7 and MCF-7/ADR cell lines

**Figure 2:**
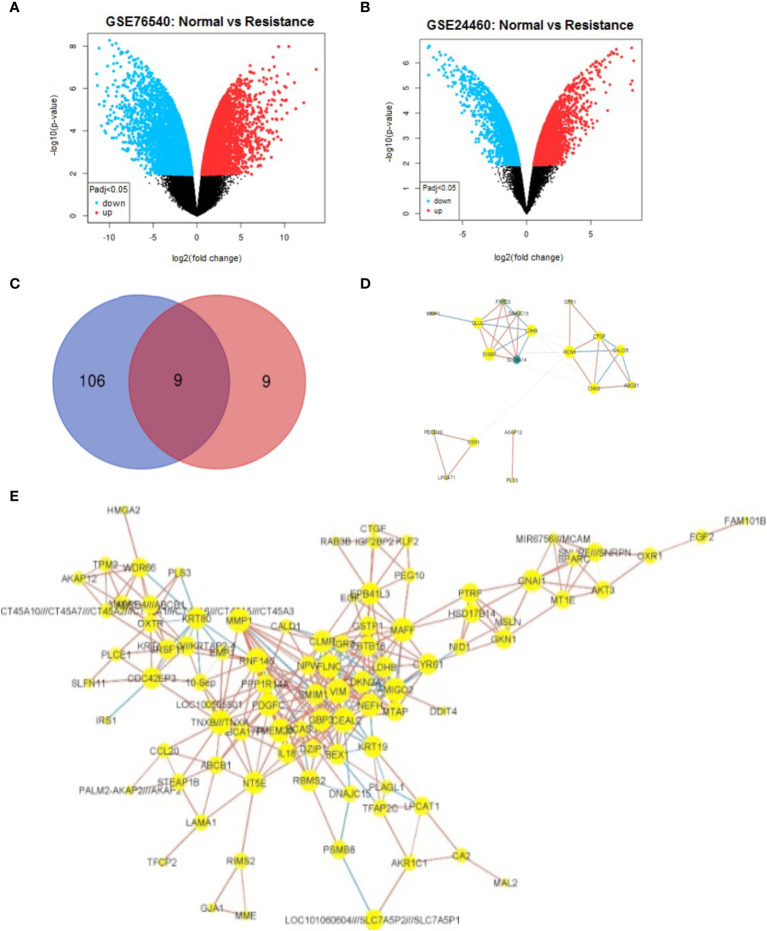
Identification and co-expression network visualization of DEGs between MCF-7 and MCF-7/ADR cell lines. Volcano plots show the overall changes in gene expression between MCF-7 and MCF-7/ADR cell lines analyzed in the GSE76540 **(A)** and GSE24460 **(B)** datasets. Among a final list of 124 DEGs, only nine were shared between the two datasets, as depicted in the Venn diagram **(C)**. One hundred and six DEGs were only detected in the GSE76540 dataset and nine in the GSE2446 dataset. The gene co-expression network of DEGs identified in the GSE24460 **(D)** and GSE76540 dataset **(E)**.

**Figure 3:**
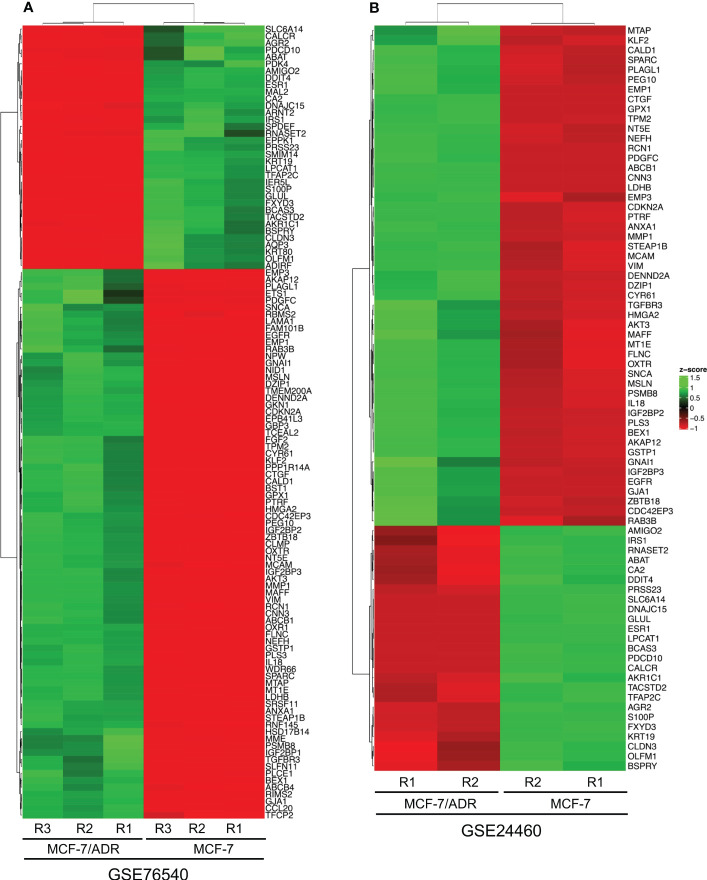
The heatmaps show the differentially expressed genes (DEGs) between the MCF-7 and MCF-7/ADR cell lines in the GSE76540 **(A)** and GSE24460 **(B)** datasets.

To identify DEGs, the microarray gene expression profiles of MCF-7/ADR and its parent MCF-7 cell line were compared in two independent GSE datasets (GSE24460 and GSE76540), considering a minimum fold change in expression > 2 and an FDR-corrected P-value cutoff of 0.05 (**Table 1**; **Supplementary Data 1**). We identified 1,108 DEGs in GSE24460 (566 up-regulated and 542 down-regulated DEGs) and 3,207 in the GSE76540 dataset (1,835 up-regulated and 1,372 down-regulated DEGs), as shown by volcano plots in **Figures 2A**, **B**. Correlating gene expressions in each data set, considering a correlation coefficient > 0.7 and an adjusted P-value < 0.05, resulted in the identification of 36 strongly co-regulated genes in GSE24460 and 406 in GSE76540. GCN analysis resulted in the identification of 18 and 115 genes with the highest degree and/or betweenness for the GSE24460 and GSE76540 datasets, respectively (**Figures 2C–E**). Among the final list of candidate co-expressed genes, only nine were shared in the two data sets, some of which are already known to contribute to chemoresistance, including *ABCB1*, *LDHB*, and *ESR1*. We thus identified a total of 122 differentially expressed genes (two genes were excluded from further analysis due to a lack of gene symbols) between MCF-7/ADR and MCF-7 cells, including 86 upregulated and 36 downregulated genes. The expression patterns of the candidate DEGs in the two datasets are visualized in heatmaps (**Figure 3**).

### Functional enrichment analysis of DEGs

**Figure 4:**
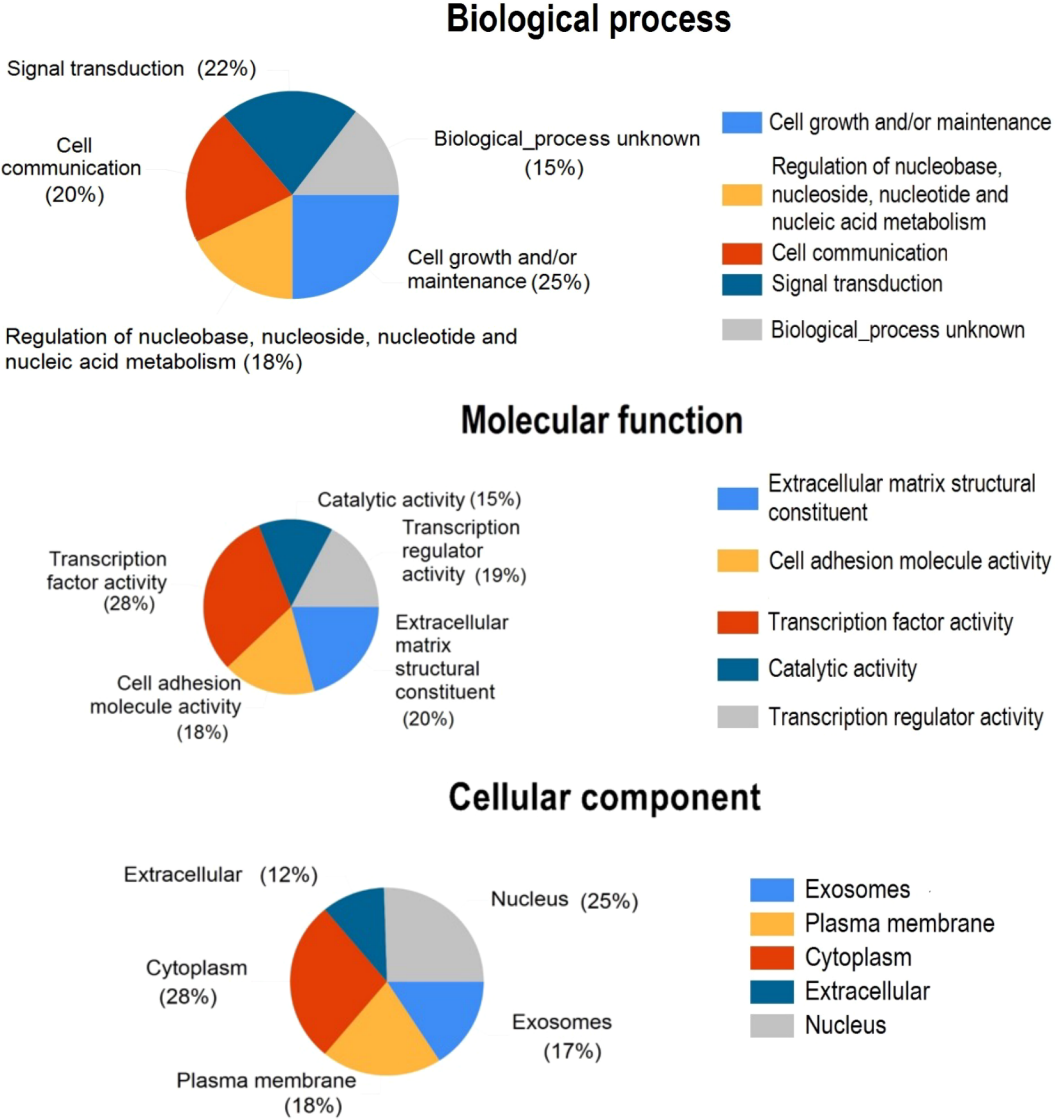
Pie charts show the gene ontology annotations for the DEGs discovered by GCN analysis. DEGs were classified as cellular components, molecular functions, and biological processes.

To link the candidate DEGs to biological or molecular processes, a gene ontology enrichment analysis was performed. The gene enrichment analysis was conducted by the FunRich GO analysis software suite (44). Genes associated with all three functional categories, including biological processes (BP), molecular processes (MP), and cellular components (CC), were identified among the candidate DEGs. The genes categorized in the CC group mostly originate from the cytoplasm, nucleus, plasma membrane, and exosome. Genes associated with the MF category were mainly transcription factors, extracellular matrix structural constituents, cell adhesion molecules, and transcription regulators. Cell growth and/or maintenance, signal transduction, and cell communication were significantly enriched in the BP group (**Figure 4**).

**Figure 5:**
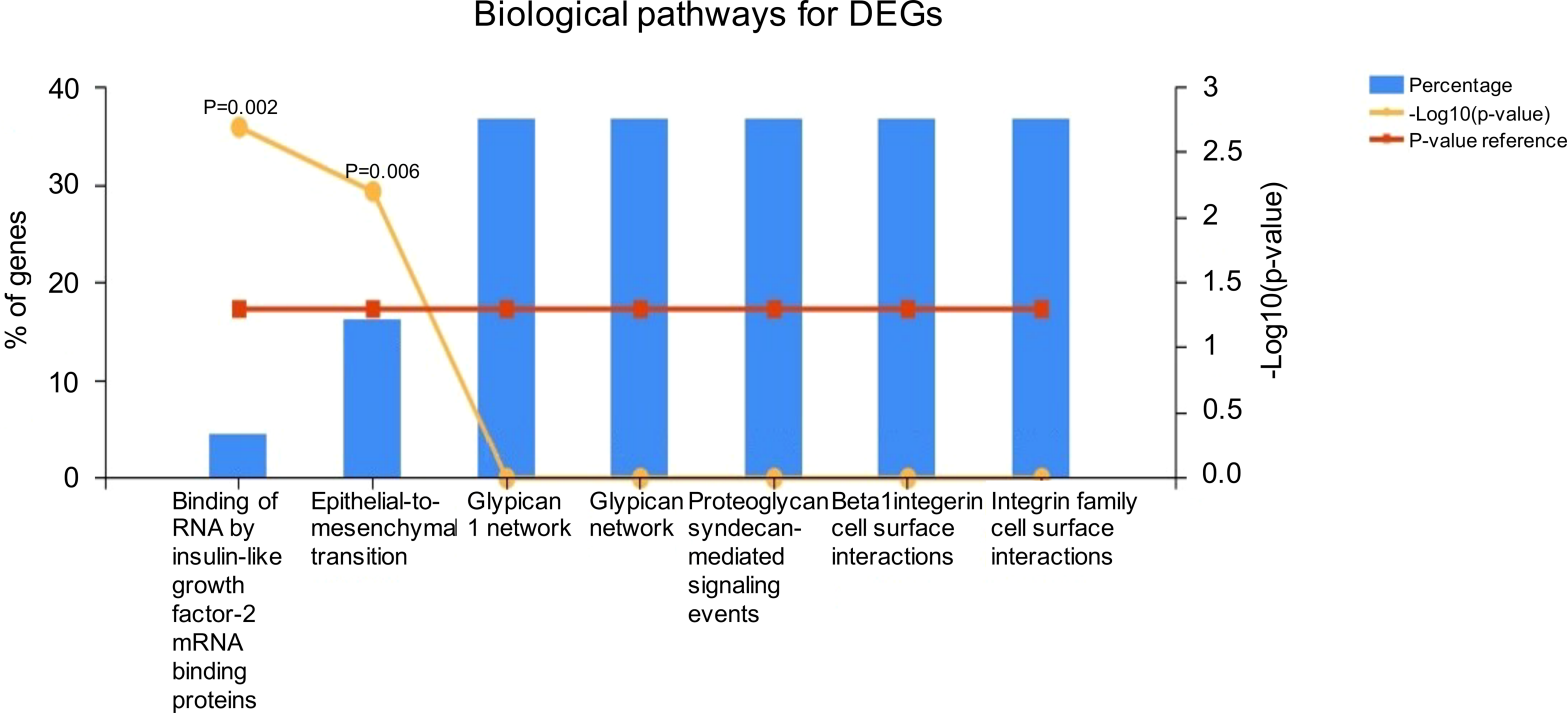
Functional enrichment analysis using the Kyoto Encyclopedia of Genes and Genomes (KEGG) database.

Most of the DEGs were enriched in pathways associated with insulin-like growth factor-2 mRNA binding proteins and epithelial-to-mesenchymal transition (**Figure 5**). KEGG pathway analysis also revealed that most of the DEGs are involved in propanoate and pyruvate metabolism, bladder cancer, and membrane transport (ABC transporters).

### Establishing a PPI network for the candidate DEGs, cluster analysis, and selection of hub genes

**Figure 6:**
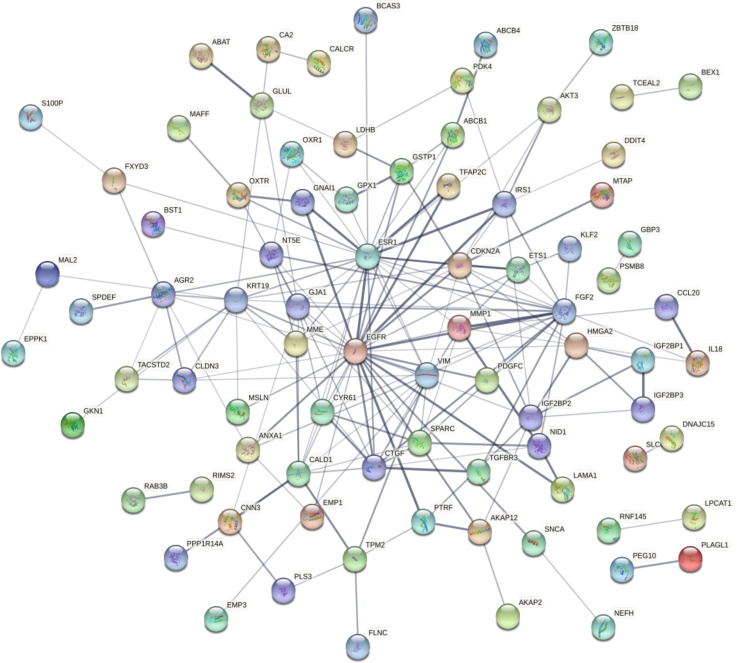
The network shows the protein-protein interactions between the candidate DEGs identified by comparing the transcriptomes of the MCF-7/ADR and MCF-7 cell lines. Proteins are represented by circles. Significant associations between proteins are shown by lines.

The candidate co-expressed genes were searched for potential protein-protein interactions using STRING. The resulting PPI network included 122 nodes and 50 edges, with a PPI enrichment P-value of 1.3e-12 (**Figure 6**). Cluster analysis using Cytoscape revealed a critical module across the network with 10 essential co-regulated genes, including *EGFR*, *ESR1*, *FGF2*, *CDKN2A*, *KRT19*, *VIM*, *CTGF*, *CALD1*, *GJA1*, and *MMP1*. Particularly, *EGFR* and *ESR1* genes showed the highest degree of connectivity in the PPI network, suggesting their critical role in maintaining the integrity of the whole network. To confirm whether the changes in gene expression detected by microarray could be validated in the corresponding cell lines, the expression of these functionally significant genes was evaluated by qRT–PCR.

### MCF-7/ADR cells showed resistance to doxorubicin-mediated apoptosis

**Figure 7:**
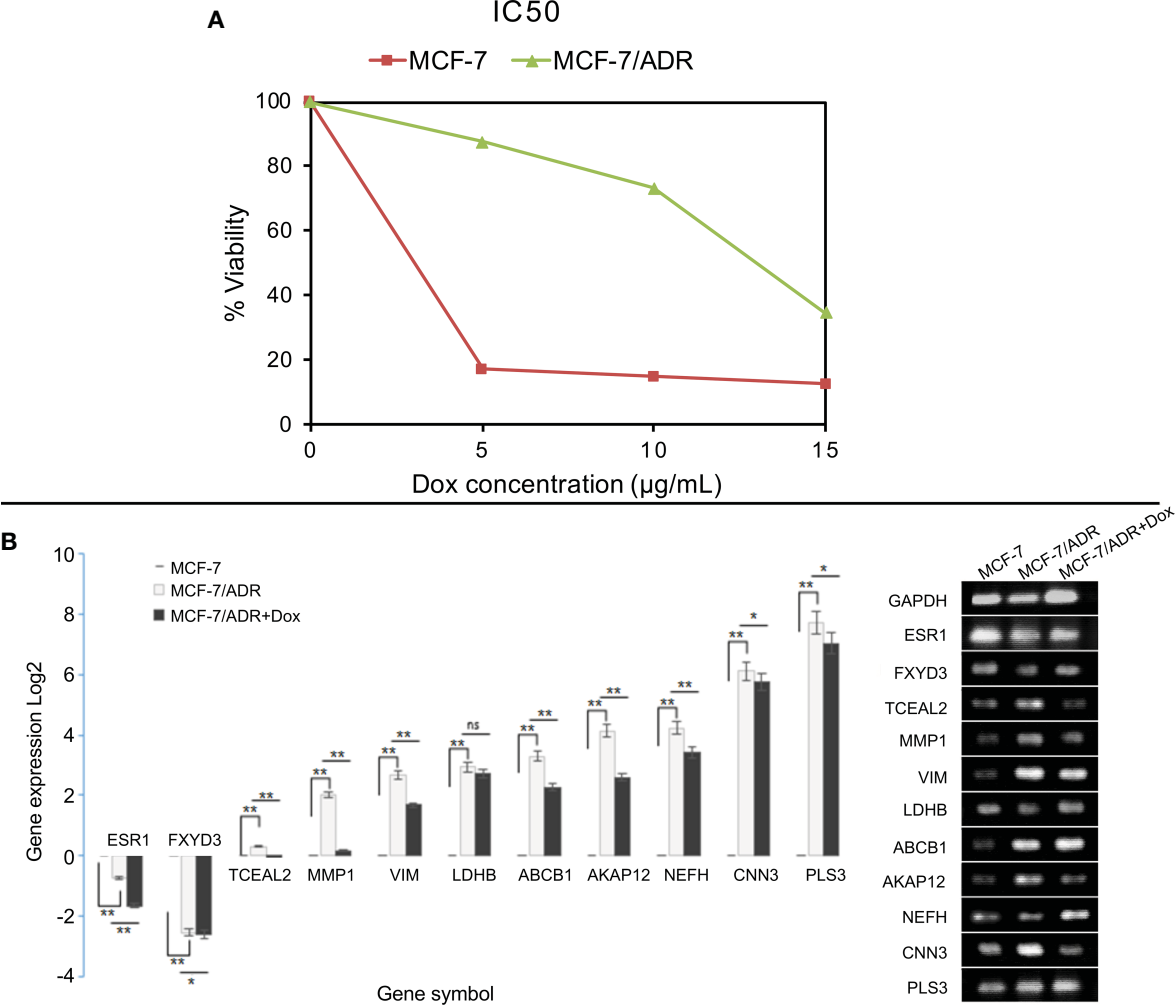
Cell viability and gene expression in the presence of doxorubicin in MCF-7/ADR and its parent cell line MCF-7. MCF-7 and MCF-7/ADR cells were treated with doxorubicin at concentrations of 0, 5, 10, and 15 μg/mL for 48 hours, and cell viability was determined using the MTT assay **(A)**. qRT–PCR analysis of the expression of candidate DEGs in MCF-7 and MCF-7/ADR in the presence or absence of doxorubicin **(B)**. The genes were selected from the GCN network based on their degree or betweenness. *CTGF* failed to amplify in both cell lines. *P-value < 0.05 and **P-value <0.01.

To characterize whether MCF-7 and MCF-7/ADR cell lines have the same genetic origin, we performed STR profiling. Our results showed that MCF-7/ADR and its parent MCF-7 cell line have a shared STR profile, confirming their common origin. To test whether our MCF-7/ADR cell line has maintained its drug resistance phenotype, cell viability under an increasing concentration of doxorubicin was evaluated. Exposing MCF-7 cells to an increasing dose of doxorubicin for up to 48 h significantly decreased cell viability, while no significant change to the MCF-7/ADR cells was noted. MCF-7/ADR cells had a vitality almost three times higher than that of their parent MCF-7 cells. Doxorubicin treatment effectively suppressed the development of MCF-7 and MCF-7/ADR cells with an IC_50_ value of 3.09 ± 0.03 and 13.2 ± 0.2 μg/mL, respectively (**Figure 7A**). Doxorubicin at doses of 5, 10, and 15 μg/mL significantly inhibited the proliferation of MCF-7 cells (P<0.05). MCF-7/ADR cells treated with 5 and 10 μg/mL of doxorubicin for 7 days displayed a normal cell morphology with only minor swelling.

### Evaluating the expression of the candidate DEGs in the MCF-7 and MCF-7/ADR cell lines

The qRT–PCR method was used to confirm the expression of ten candidate co-expressed genes, five of which were shared between the two datasets, namely *MMP1*, *ABCB1*, *AKAP12*, *PLS3*, and *CTGF*. Three genes were selected from the GSE76540 dataset, namely *VIM*, *TCEAL2*, and *NEFH*, while two genes were selected from the GSE24460 dataset, including *LDHB* and *CNN3* (**Table 3**). Two additional co-downregulated genes, ESR1 and FXYD3, were also included to further check for amplification biases. The expression of genes was compared between MCF-7 and MCF-7/ADR cell lines in the presence or absence of doxorubicin. In the absence of the drug, a low expression of *ESR1* and *FXYD3* mRNAs in MCF-7/ADR cells was noticeable. Their expressions increased steadily in the presence of the drug at concentrations greater than 5 μg/mL. A significant difference in *PLS3*, *CNN3*, and *NEFH* expression was also noted between MCF-7 and MCF-7/ADR cells, correlating with microarray data (**Figure 7B**). *CTGF* was the only gene for which no expression was detected by qRT–PCR.

**Table 3:** The list of the candidate up/down-regulated genes selected for qRT-PCR analysis following GCN analysis in the MCF-7/ADR and MCF-7 cell lines.

Data Set	Name	Rank_stat	Degree	Betweenness
GSE76540	*VIM*	111.0	22	408.1
	*TCEAL2*	112.0	21	549.4
	*NEFH*	103.25	18	289.2
GSE24460	*LDHB*	15.5	7	10.9
	*CNN3*	13.5	6	5.8
GSE76540 and GSE24460	*MMP1*	107.75	18	391.8
	*LDHB*	15.5	7	10.9
	*ABCB1*	70.25	7	70.9
	*AKAP12*	43.25	4	0.4
	*PLS3*	27.25	3	0.0
	*CTGF*	11.25	5	3.8

## Discussion

Doxorubicin is widely used as a first-line neoadjuvant chemotherapy medication to treat breast cancer (45). While doxorubicin therapy proved to be extremely effective in the short-term treatments, long-term use may result in chemoresistance. Resistance to doxorubicin is a significant obstacle to the effectiveness of chemotherapy in patients with breast cancer. While the mechanism of chemoresistance is complex, identifying the critical genes and signaling pathways involved in the process is practically challenging. Large-scale analysis of gene expression in chemo-sensitive and chemo-resistant cells is a key approach to identifying genes or pathways associated with this phenomenon.

Here, we sought to explore the available microarray gene expression data sets to identify potentially important components of the chemoresistance mechanisms in MCF-7/ADR cells challenged with the chemotherapeutic agent doxorubicin. GCN analysis is commonly used to identify genes or molecular pathways in complex gene expression data (46–48). Using this approach, we identified several candidate DEGs that are known to implicate cell proliferation and/or maintenance, insulin-like growth factor 2 mRNA binding, and epithelial-to-mesenchymal transition (EMT). These include several potential hub genes in the PPI network, such as *EGFR*, *ESR1*, *FGF2*, *CDKN2A*, *VIM*, *CTGF*, *CALD1*, and *MMP1*. Only *ESR1* and *FXYD3* showed decreased expression in the chemo-resistant cell line, whereas the remaining genes showed upregulation.

The most common mechanism for chemoresistance is the active efflux of the chemotherapeutic agent through the ABC transporters (28). ABC transporters are a large group of membrane proteins that mediate the import or export of diverse substrates across the cell membrane (49). Overexpression of cell surface efflux ABC transporters, including *ABCB1*, *ABCC1*, and *ABCG2*, was associated with chemoresistance in breast cancer (50, 51). Our analysis showed an increased expression of ABCB1, indicating its potential role in conferring resistance to doxorubicin.

Lactate dehydrogenase B (LDH-1) is a glycolytic enzyme linked to lysosomes and autophagy via the oxidative pathway (52, 53). Deacetylation of LDHB by SIRT5 promotes the development of autophagy vesicles and thus induces autophagy (54). Breast cancer cells expressing high levels of *LDHB* show basal-like and glycolytic phenotypes, whereas the suppression of its expression reduces their glycolytic dependence (55). The expression of *LDHB* is significantly increased in response to chemotherapy, suggesting a marker role for this gene in response to neoadjuvant chemotherapy in breast cancer (56). In line with our results, previous proteomic analysis of Adriamycin resistance in breast cancer also suggested a role for *LDHB* in drug resistance (57).

*PLS3* encodes a protein called plastin-3 that is found in cancer cells. It promotes apoptosis via the TRAIL pathway, which is accomplished by expanding the death pathway of the mitochondrial arm (58). However, *PLS3* has been identified as a putative target for increasing p38 MAPK-mediated apoptosis triggered by drug resistance, suggesting that targeting this enzyme could be an effective strategy for overcoming drug resistance (59). We identified *AKAP12* as a key component of chemoresistance in the MCF-7/ADR cell line. In ovarian cancer, *AKAP12* has been associated with paclitaxel resistance by modulating signaling pathways related to cell survival and drug efflux (60). The role of this protein in doxorubicin tolerance is not well understood.

*MMP-1* is a gene that encodes a zinc-dependent matrix-metalloprotease involved in the activation of EMT, the Akt signaling pathway (61), and angiogenesis through various mechanisms (62). Snail, Slug, and Twist are EMT-promoting transcription factors that directly induce *MMP-1* transcription in chemo-resistant cells (63). By inactivating the Fas receptor, *MMP-1* suppresses apoptosis and increases chemoresistance (64). In addition, other members of the MMP family proteases, including MMP-2 (65), MMP-7 (66), and MMP-9 (67), also contribute to metastasis and multidrug resistance by degrading extracellular matrix components in multiple types of cancer. MMP-1 expression has been linked to increased cell proliferation, tumor development, metastasis, and resistance to chemotherapy in various tumors (68, 69). Several studies have shown that overexpression of *MMP-1* significantly reduced drug sensitivity in MCF-7 cells, whereas *MMP-1* knockdown considerably increased drug sensitivity in MCF-7/ADR cells (63, 70). Another candidate DEG that is known to be involved in EMT is vimentin (VIM). The contribution of VIM to EMT is mediated by activating the Akt signaling pathway (71). CNN3 (calponin-3) expression is altered in colorectal and breast carcinomas (72, 73). It has been linked to EMT via β-Catenin, ERK1/2, c-Jun, heat shock protein 60, and mutant p53 pathways (74).

Our results suggest that gene co-expression network analysis can be used to identify genes that contribute to doxorubicin resistance in breast cancer. Several of these genes have been associated with cancer development, progression, metastasis, and resistance to chemotherapy based on previous studies. In many cases, potential inhibitors have been identified that could be used to overcome drug resistance (75, 76). This research included some limitations. For instance, it took advantage of microarray data; however, the results can be further complemented by additional analyses using mRNA sequencing and proteomics data to characterize changes in protein abundances, post-translational modifications, and protein localizations. Additional functional analyses, including gene knockout and/or overexpression, can help provide a mechanistic understanding of the role of these genes in the development of chemoresistance in breast cancer.

## Conclusion

In this study, we analyzed microarray data sets from chemo-resistant and chemo-sensitive breast cancer cell lines using GCNs. Our results suggest a key role for certain extracellular matrix component proteins in the development of chemoresistance in the MCF-7/ADR breast cancer cell line. The results of the bioinformatics analysis were confirmed by qRT–PCR analyses of specific DEGs. These findings could pave the way for the identification of genes linked to molecular mechanisms governing chemotherapy resistance in breast cancer.

## Data availability statement

The datasets presented in this study can be found in online repositories. The names of the repository/repositories and accession number(s) can be found in the article/**Supplementary Material**.

## Author contributions

AM performed the experiments, analyzed the data, and drafted the manuscript. JG contributed to the writing and revision of the manuscript. ISM conducted the analysis of gene expression data. KS and VJ conceived and designed the study, and contributed to the writing of the manuscript. All authors reviewed and approved the final version of the manuscript.

## Supplementary material

Supplementary Data Sheet 1 The complete list of all differentially expressed genes (DEGs) in GSE76540 and GSE24460, as determined by contrasting the MCF-7/ADR and MCF-7 cell lines. Statistically significant expression was defined as having a LogFC > 1 and an adjusted P-value cutoff of 0.05.
